# Supplementary figures and images for: High-throughput analysis of adaptation using barcoded strains of Saccharomyces cerevisiae
Source: PeerJ. 2020 Oct 16;8:e10118. doi: 10.7717/peerj.10118 (PMC7571412; doi:10.7717/peerj.10118)

A

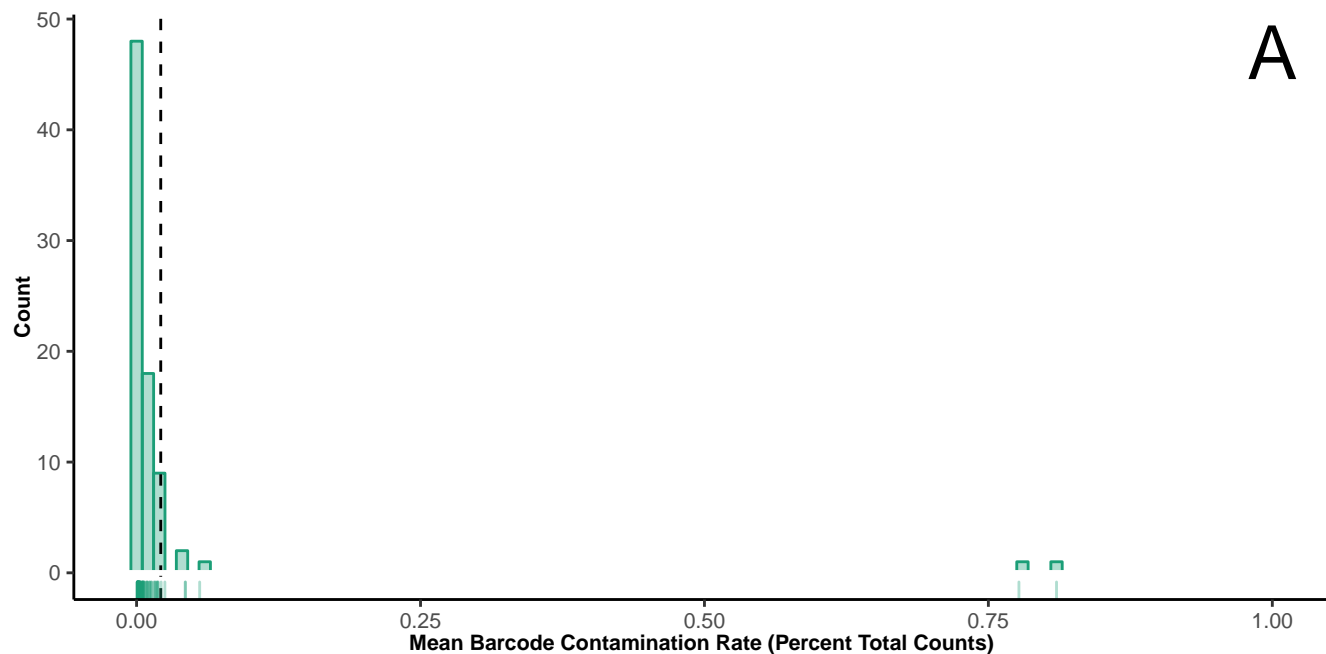

B

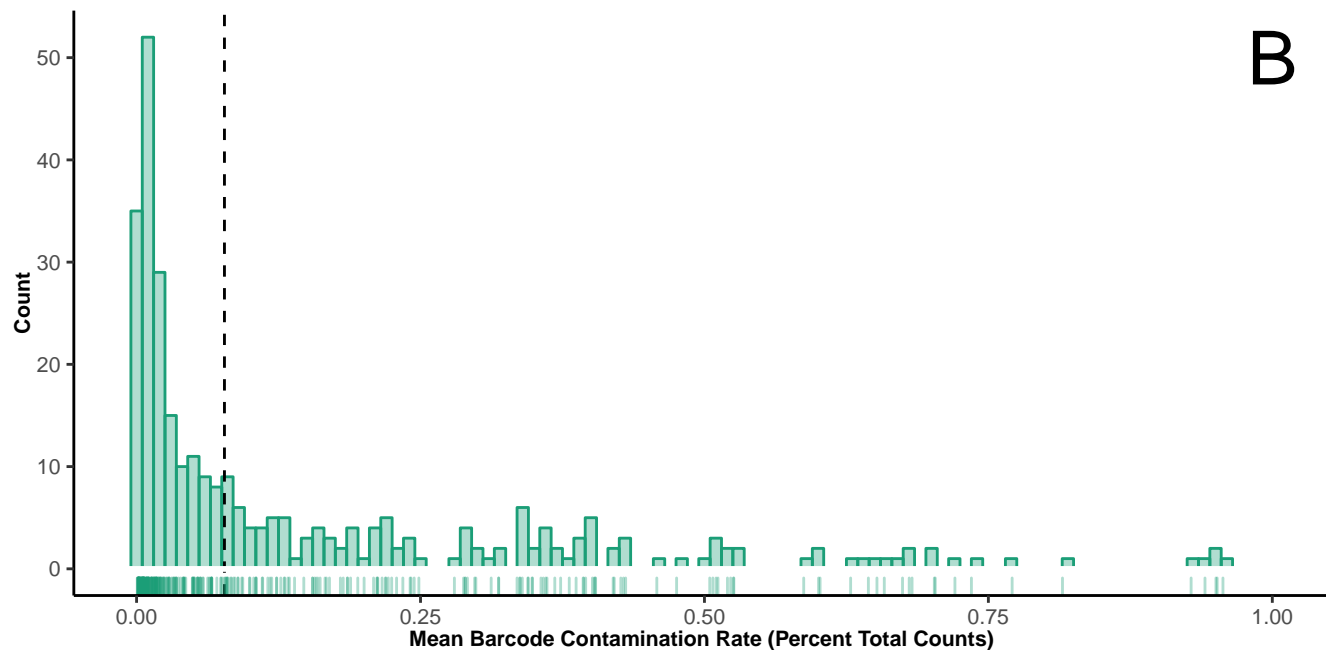

Supplement: Figure S1 — A. Histogram of mean barcode contamination rate (as a percentage of total count) for the cross-contamination diagnostic samples included in the Proof of Concept Fitness Assays sequenced library. One datapoint is reported for each unique forward-reverse index pair (sample) in the sequenced library. B. Histogram of mean barcode contamination rate (as a percentage of total count) for samples included in the 250-generation experimental evolution project. For resequenced samples, only the less contaminated sample is retained. One datapoint is reported for each unique forward-reverse index pair (sample) in this consensus library. [file peerj-08-10118-s001.pdf]

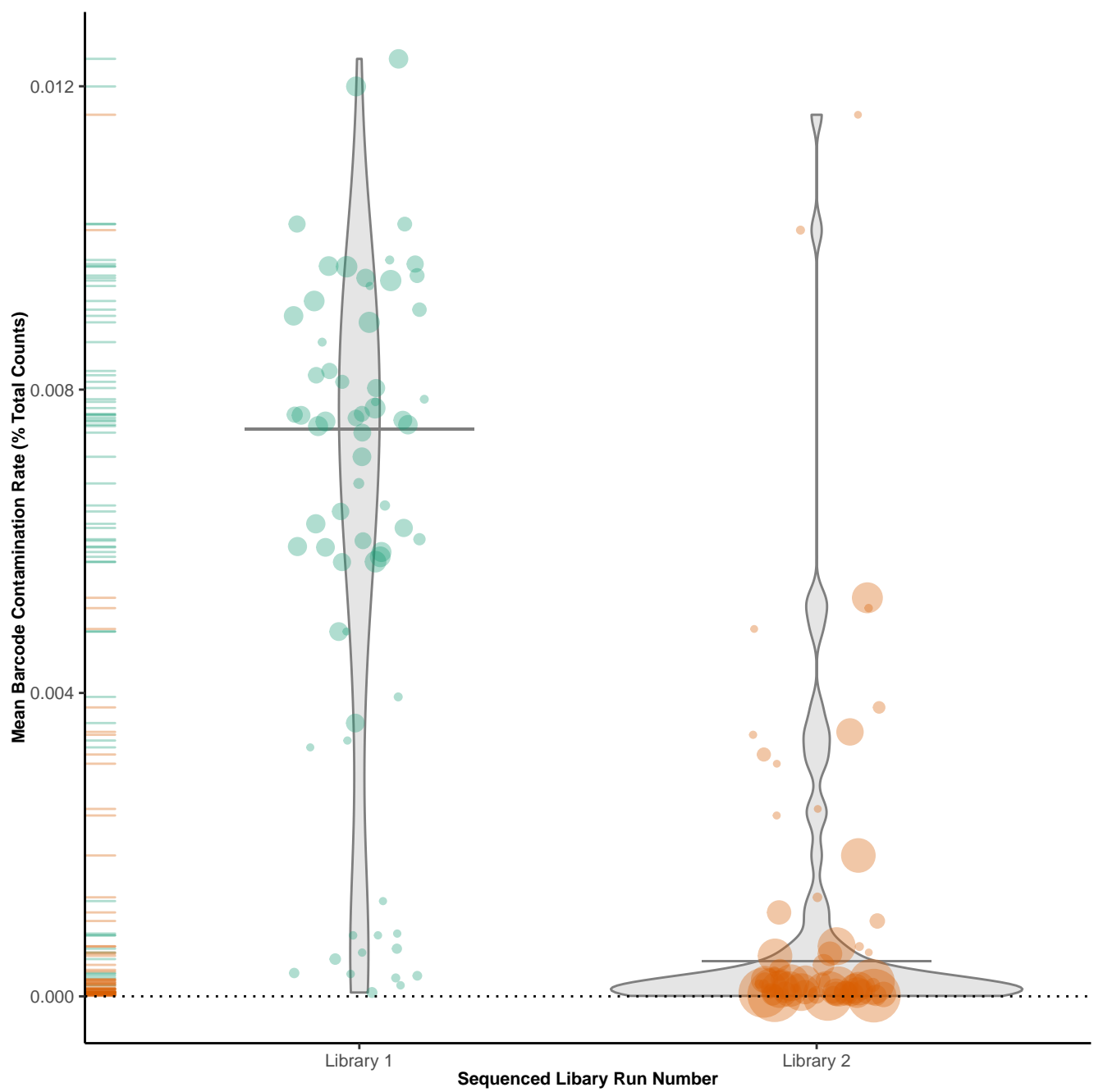

Supplement: Figure S2 — Violin plots showing decrease in barcode contamination rate for a set of samples from the 250-generation evolution experiment that were DNA extracted, PCR amplified, and sequenced two separate times. Colors depict sequencing runs: cyan for run 1., and orange for run 2. Point sizes reflect the number of reads underlying each datapoint. Mean contamination rate for run 1., and run 2., are depicted as heavy black crossbars. [file peerj-08-10118-s002.pdf]

Treatment

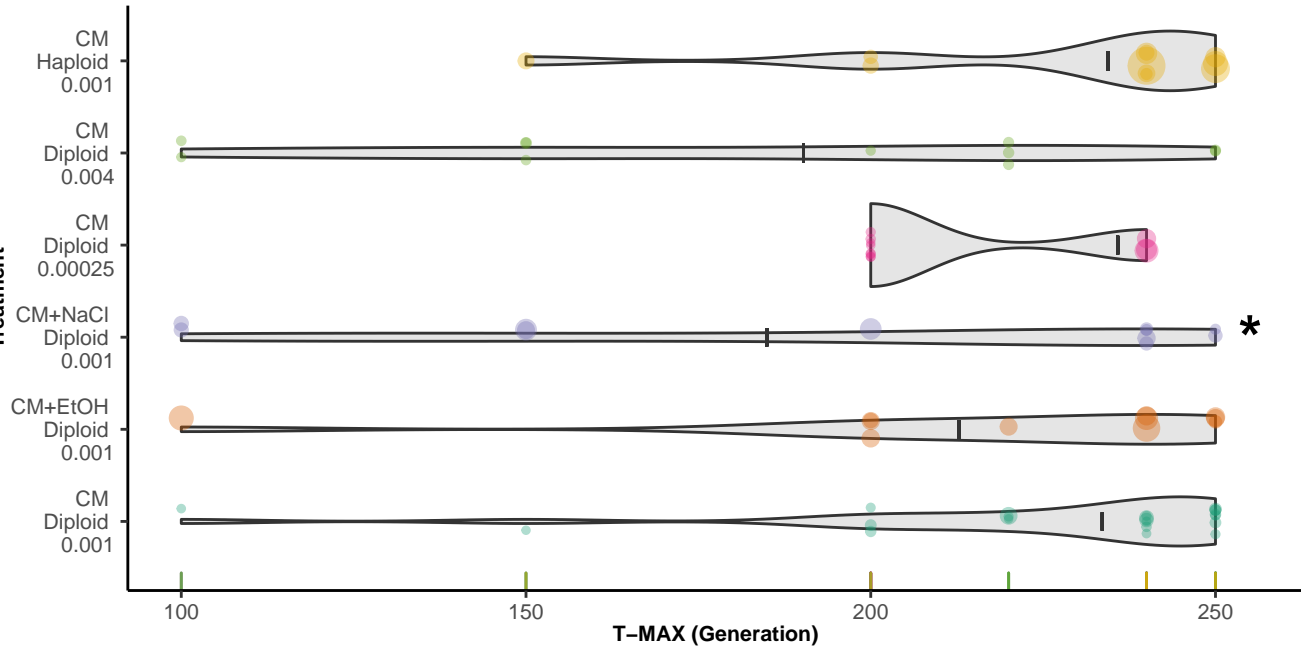

Supplement: Figure S3 — Violin plot of generation of maximum deviation in barcode abundance from initial conditions for 152 yeast strains evolved across six evolutionary treatments for 250 generations. Point sizes reflect the number of reads underlying each datapoint and colors indicate evolutionary treatments. Treatment means are depicted as heavy black crossbars. Treatments significantly different from the control treatment are marked with an asterisk. The treatment with diploid yeast evolved under a standard 1:1000 transfer dilution in CM is selected as the reference level in this model. [file peerj-08-10118-s003.pdf]

Treatment

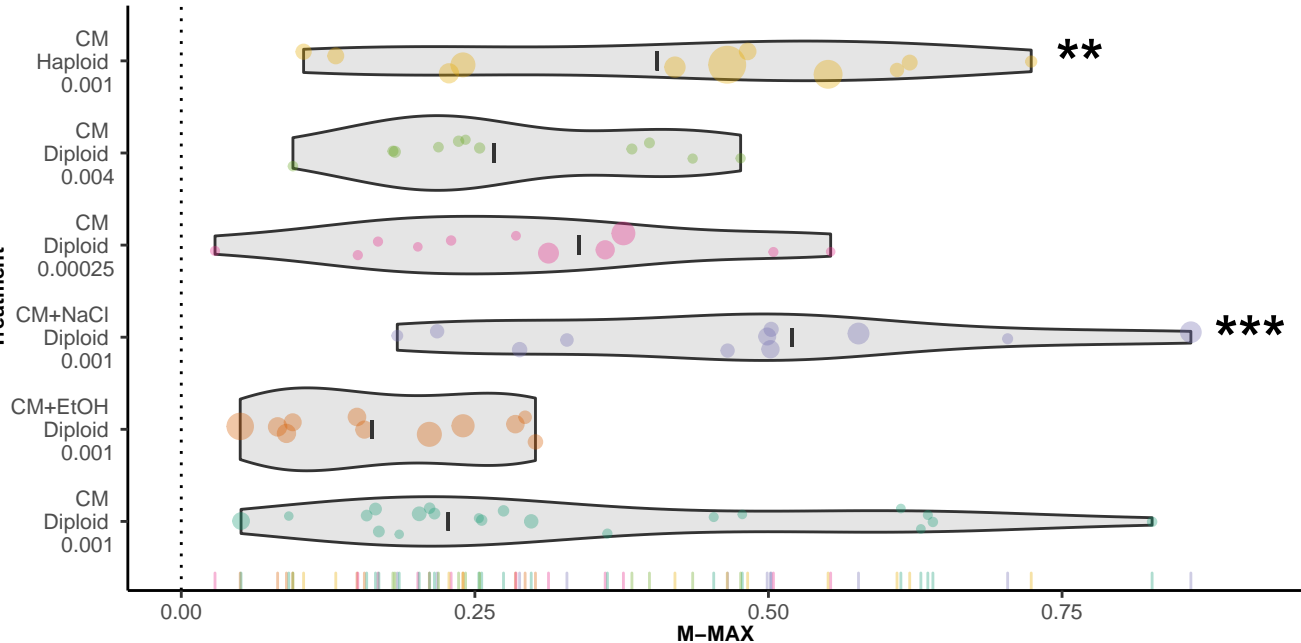

Supplement: Figure S4 — Violin plot of magnitude of maximum deviation in barcode abundance from initial conditions for 152 yeast strains evolved across six evolutionary treatments for 250 generations. Point sizes reflect the number of reads underlying each datapoint and colors indicate evolutionary treatments. Treatment means are depicted as heavy black crossbars. Treatments significantly different from the control treatment are marked with an asterisk. The treatment with diploid yeast evolved under a standard 1:1000 transfer dilution in CM is selected as the reference level in this model. [file peerj-08-10118-s004.pdf]

Treatment

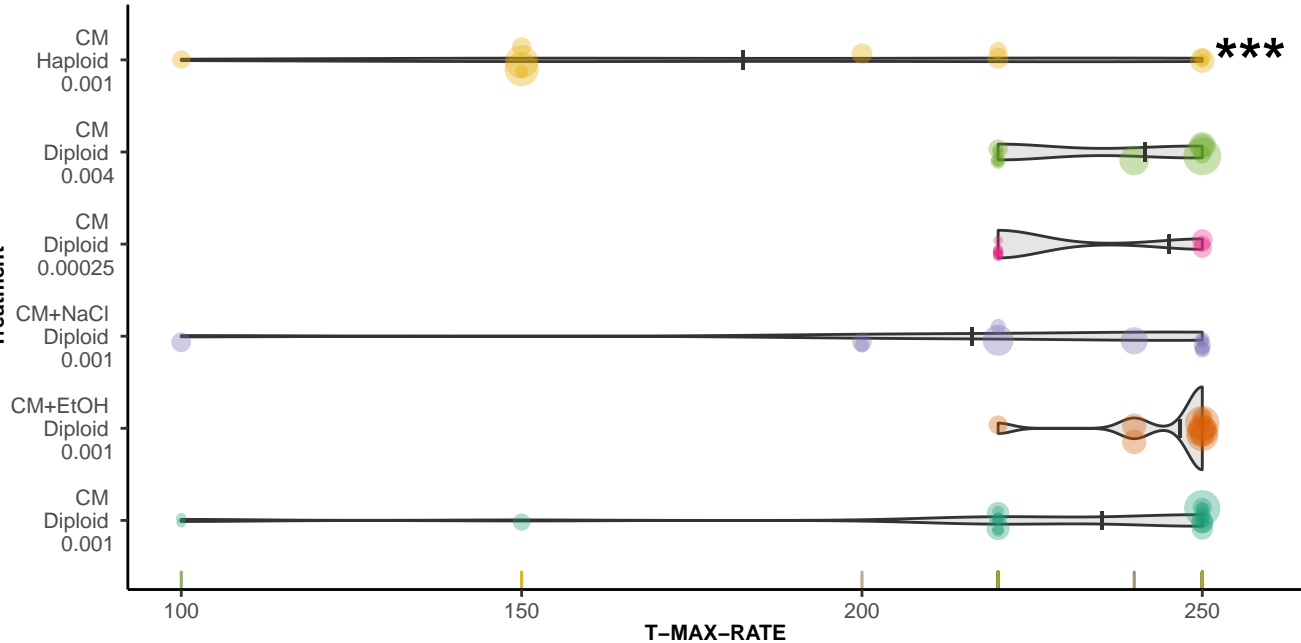

Supplement: Figure S5 — Violin plot of generation of maximum rate of change in barcode abundance for 152 yeast strains evolved across six evolutionary treatments for 250 generations. Point sizes reflect the number of reads underlying each datapoint and colors indicate evolutionary treatments. Treatment means are depicted as heavy black crossbars. Treatments significantly different from the control treatment are marked with an asterisk. The treatment with diploid yeast evolved under a standard 1:1000 transfer dilution in CM is selected as the reference level in this model. [file peerj-08-10118-s005.pdf]

Treatment

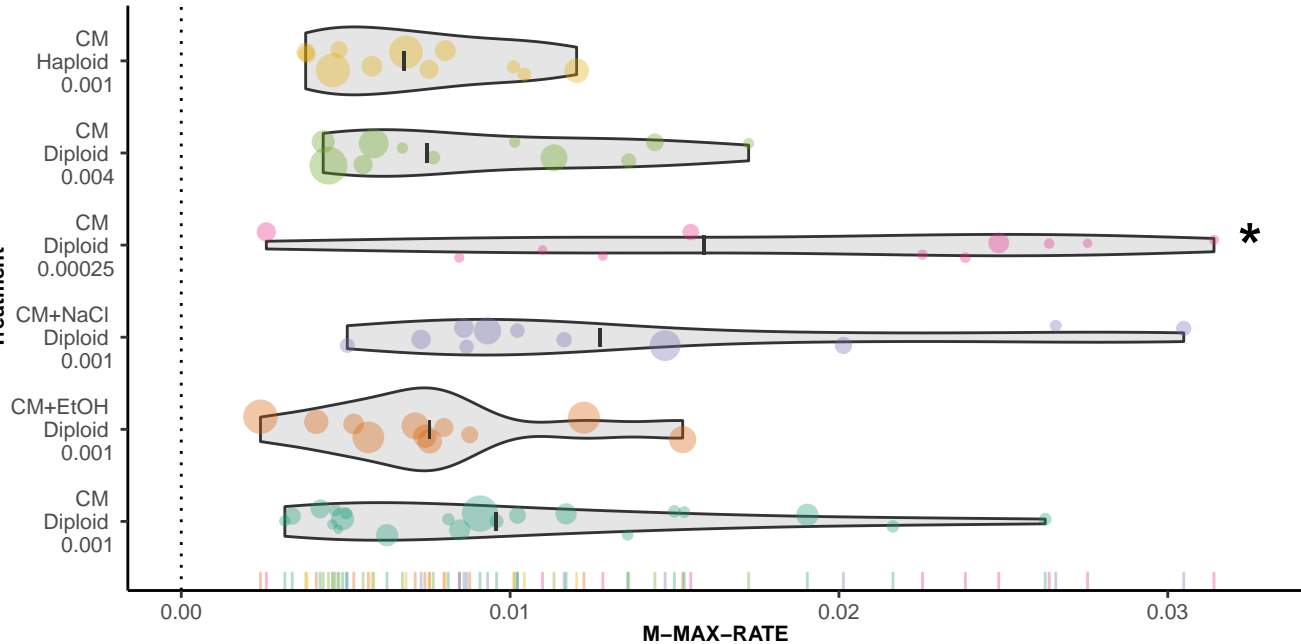

Supplement: Figure S6 — Violin plot of magnitude of maximum rate of change in barcode abundance for 152 yeast strains evolved across six evolutionary treatments for 250 generations. Point sizes reflect the number of reads underlying each datapoint and colors indicate evolutionary treatments. Treatment means are depicted as heavy black crossbars. Treatments significantly different from the control treatment are marked with an asterisk. The treatment with diploid yeast evolved under a standard 1:1000 transfer dilution in CM is selected as the reference level in this model. [file peerj-08-10118-s006.pdf]

Treatment

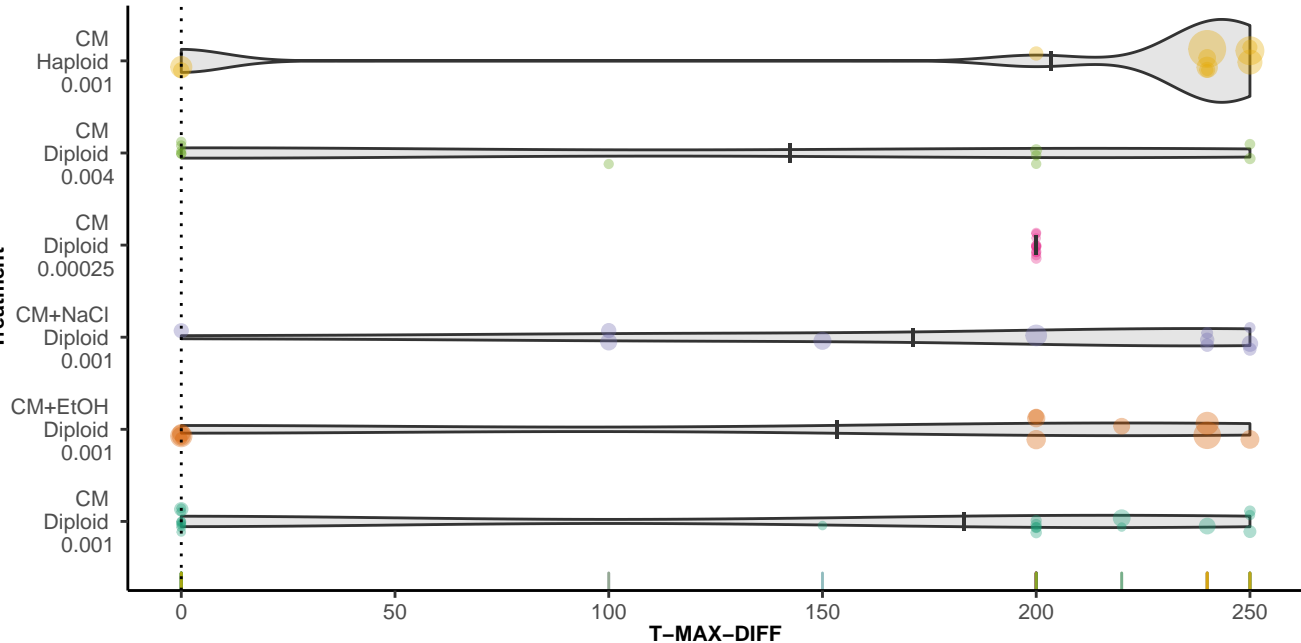

Supplement: Figure S7 — Violin plot of generation of maximum difference in sympatric barcode abundance for 152 yeast strains evolved across six evolutionary treatments for 250 generations. Point sizes reflect the number of reads underlying each datapoint and colors indicate evolutionary treatments. Treatment means are depicted as heavy black crossbars. Treatments significantly different from the control treatment are marked with an asterisk. The treatment with diploid yeast evolved under a standard 1:1000 transfer dilution in CM is selected as the reference level in this model. [file peerj-08-10118-s007.pdf]

Treatment

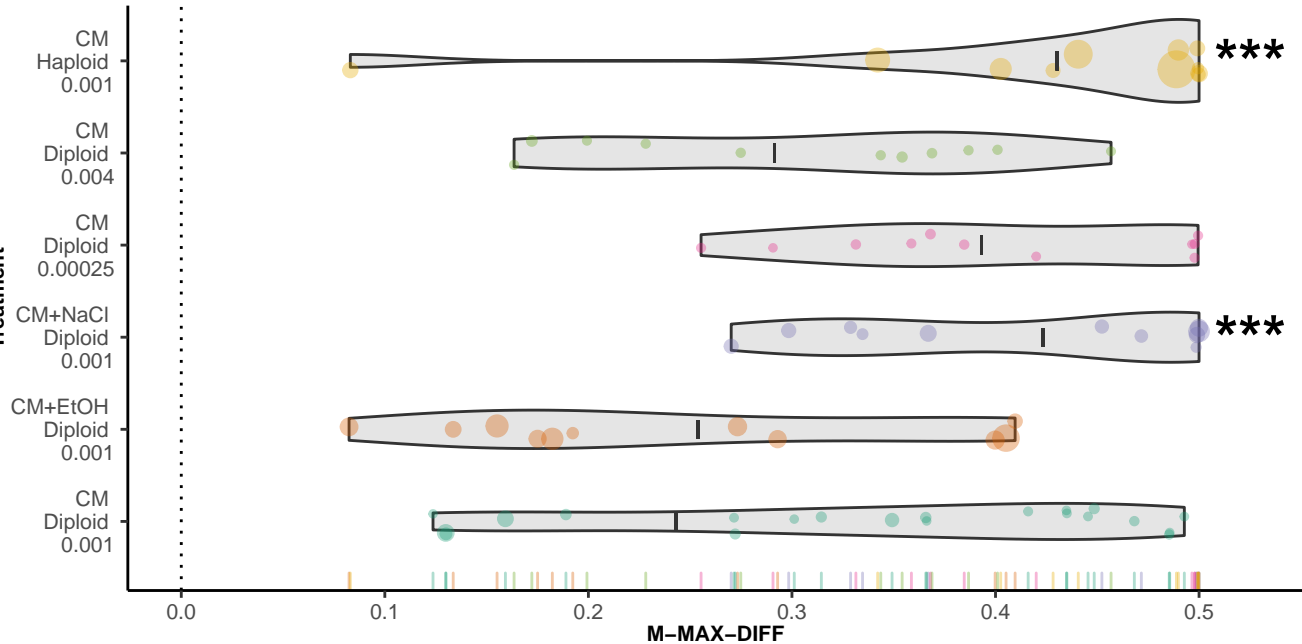

Supplement: Figure S8 — Violin plot of magnitude of maximum difference in sympatric barcode abundance for 152 yeast strains evolved across six evolutionary treatments for 250 generations. Point sizes reflect the number of reads underlying each datapoint and colors indicate evolutionary treatments. Treatment means are depicted as heavy black crossbars. Treatments significantly different from the control treatment are marked with an asterisk. The treatment with diploid yeast evolved under a standard 1:1000 transfer dilution in CM is selected as the reference level in this model. [file peerj-08-10118-s008.pdf]

Treatment

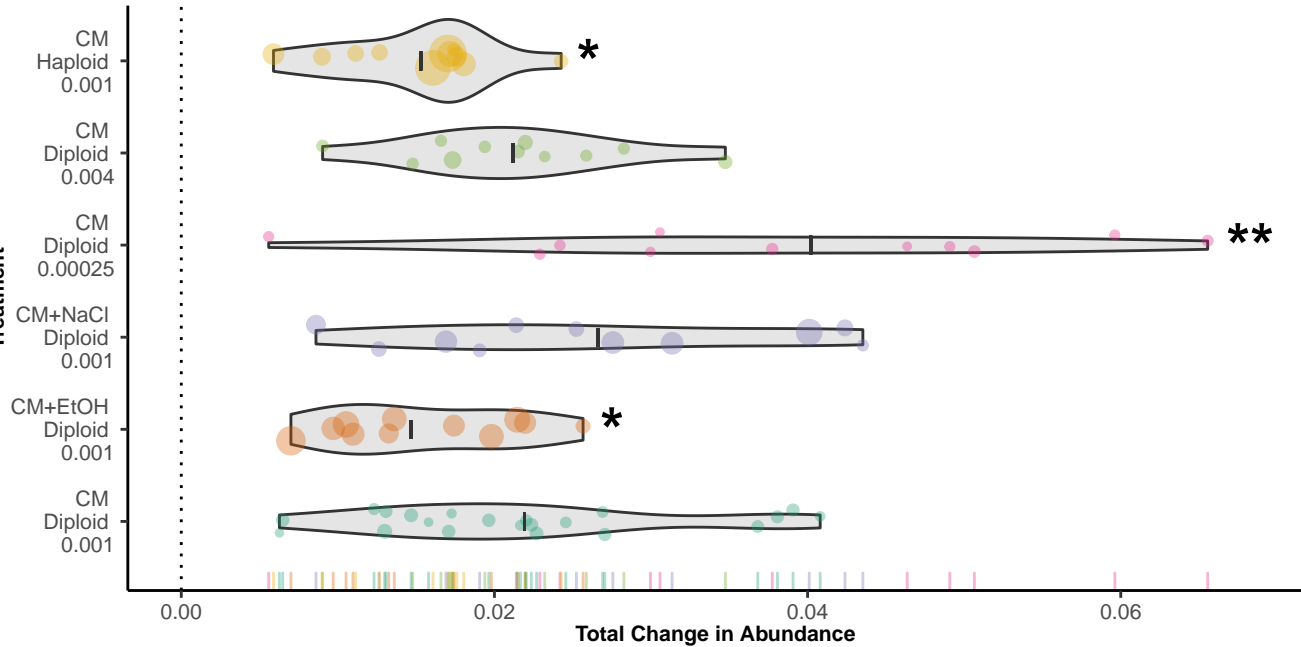

Supplement: Figure S9 — Violin plot of total (cumulative) change in sympatric barcode abundance for 152 yeast strains evolved across six evolutionary treatments for 250 generations. Point sizes reflect the number of reads underlying each datapoint and colors indicate evolutionary treatments. Treatment means are depicted as heavy black crossbars. Treatments significantly different from the control treatment are marked with an asterisk. The treatment with diploid yeast evolved under a standard 1:1000 transfer dilution in CM is selected as the reference level in this model. [file peerj-08-10118-s009.pdf]
