# Supplementary material for: High-throughput analysis of adaptation using barcoded strains of Saccharomyces cerevisiae
Source: PeerJ. 2020 Oct 16;8:e10118. doi: 10.7717/peerj.10118 (PMC7571412; doi:10.7717/peerj.10118)
Supplement: Tabele S2 — Treatment ID is a numerical ID for each of six treatments; Evo. Wells is the number of populations assigned to each treatment; Barcodes per Evo. Well is the number of sympatric barcodes per evolutionary population; Ploidy, Evo. Medium, and Evo. Transfer Dilution indicate the yeast strain ploidy, evolutionary medium type, and daily passage dilution for each treatment, respectively. [file peerj-08-10118-s011.docx]

| ***Treatment ID*** | ***Evo. Wells*** | ***Barcodes per Evo. Well*** | ***Ploidy*** | ***Evo. Medium*** | ***Evo. Transfer Dilution*** |
| --- | --- | --- | --- | --- | --- |
| 1 | 21 | 2 | Diploid | CM | 1/1000 |
| 2 | 11 | 2 | Haploid | CM | 1/1000 |
| 3 | 11 | 2 | Diploid | CM | 1/250 |
| 4 | 11 | 2 | Diploid | CM | 1/4000 |
| 5 | 11 | 2 | Diploid | CM + 8% EtOH | 1/1000 |
| 6 | 11 | 2 | Diploid | CM + 0.342M NaCl | 1/1000 |
